# Supplementary figures and images for: Single-cell atlas of the human neonatal small intestine affected by necrotizing enterocolitis
Source: PLoS Biol. 2023 May 19;21(5):e3002124. doi: 10.1371/journal.pbio.3002124 (PMC10234541; doi:10.1371/journal.pbio.3002124)

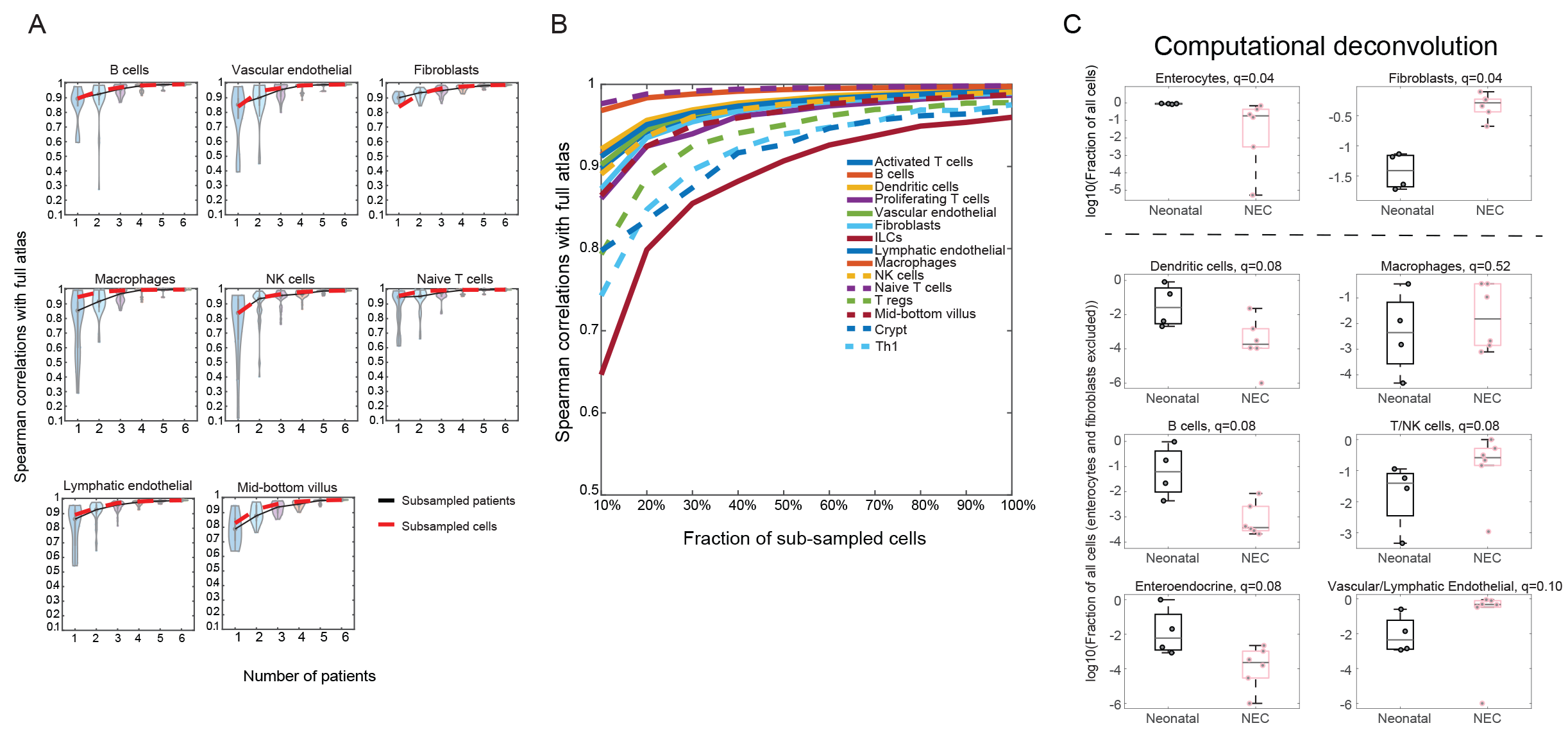

Supplement: S1 Fig — (A, B) Expression signatures of the NEC cell populations are stable with respect to number of cells and number of patients sampled. (A) Spearman correlations between the expression signatures of cell types between the full atlas and the atlas obtained by subsampling patients (black) or equal-sized groups of cells regardless of patients (red). Each subsampling contains all combinations of patients with additional 3 bootstrap iterations for each (Methods). Included are only cell types that were represented by 10 or more cells in at least 4 patients. (B) Median Spearman correlations of the complete atlas and the one obtained by sampling the indicated fractions of cells. Each value is a median over 20 bootstrap iterations. (C) Log10 scaled estimates of the proportion of enterocytes, fibroblasts, dendritic cells, macrophages, B cells, T-NK cells, enteroendocrine cells, and vascular/lymphatic endothelial cells based on computational deconvolution of the bulk RNAseq using the atlas single-cell populations (Methods). Each dot is a sample, fractions of enterocytes and fibroblasts normalized to the sum of cell fractions, remaining fractions normalized to the sum of all cells after excluding fibroblasts and enterocytes. Values are log10 (normalized data+10−6); q-values are computed based on FDR correction for all cell populations in the full atlas (Methods). Gray lines are medians, black/pink boxes are 25–75 percentiles. Only samples with Spearman correlations >0.3 between the mixture data and the synthetic mixtures are shown (neonatal: n = 4, NEC: n = 6). The data underlying this figure is available at the Zenodo repository under the following: https://doi.org/10.5281/zenodo.5813397 and in S10 Table. (TIF) [file pbio.3002124.s001.tif]

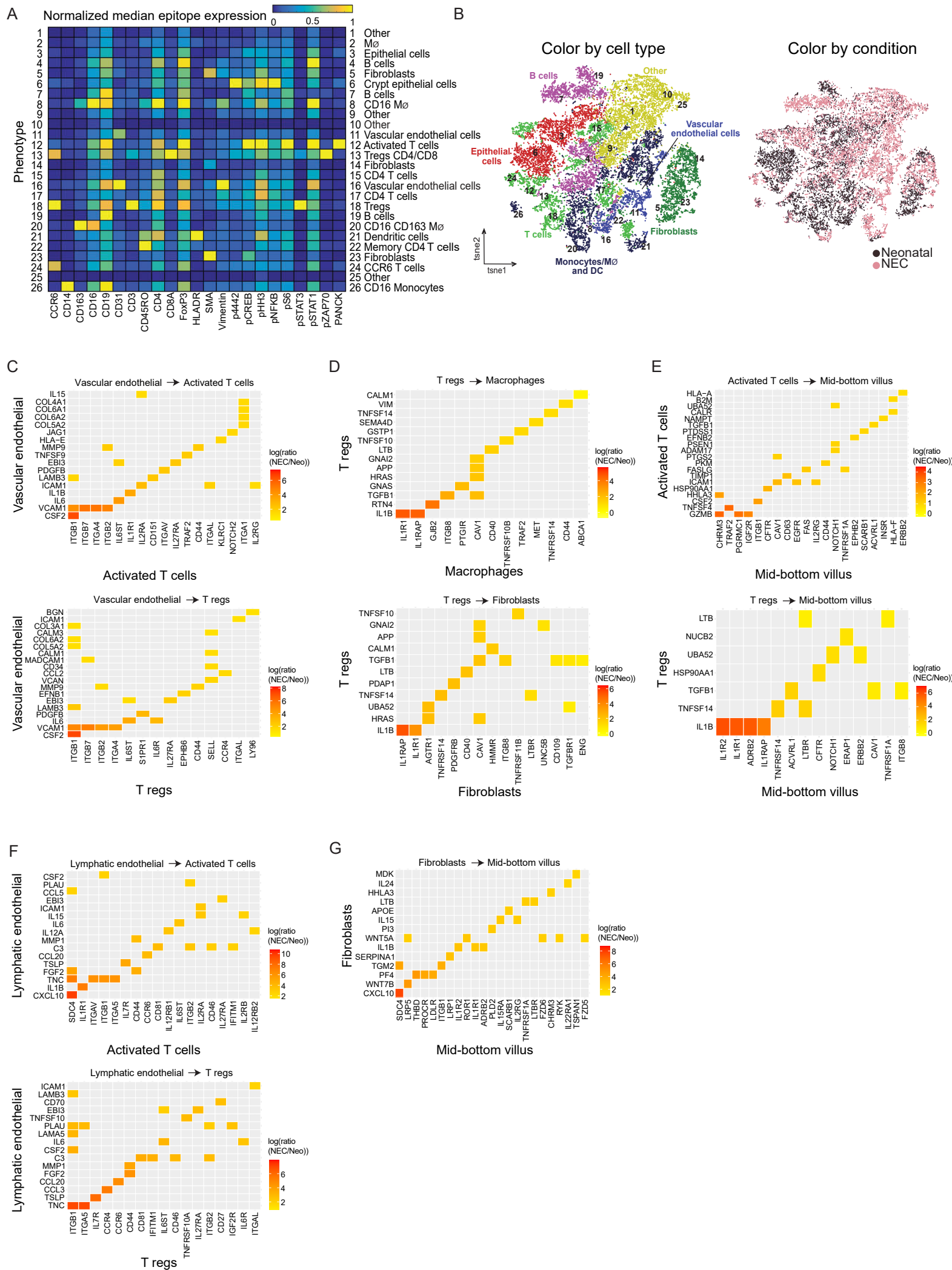

Supplement: S5 Fig — (A) Heatmap of normalized median expression of surface and phosphoprotein markers used to identify populations in IMC analysis (Methods, neonatal n = 3, NEC n = 6) depicted in B. (B) t-stochastic neighborhood embedding (tSNE) of IMC data clustered by RPhenograph and color coded by cluster (left-hand side) and condition (right-hand side). (C–G) Significantly elevated ligand–receptor interactions between cell populations explored. Shown are the log-ratios between the interaction potentials in NEC and neonatal samples (Methods). Maps include the 25 significant interactions (q-value <0.01) with highest fold change (Methods). In all interaction, maps sender population is on the y-axis, receiver population is on the x-axis. (C–G) Neo = Neonatal. The data underlying this figure is available at the Zenodo repository under the following: https://doi.org/10.5281/zenodo.5813397 and in S6 and S7 Tables. (PDF) [file pbio.3002124.s005.pdf]
