# Supplementary material for: Single-cell atlas of the human neonatal small intestine affected by necrotizing enterocolitis
Source: PLoS Biol. 2023 May 19;21(5):e3002124. doi: 10.1371/journal.pbio.3002124 (PMC10234541; doi:10.1371/journal.pbio.3002124)
Supplement: Attachment — Submitted filename: response_to_comments_ver5_LK.docx [file pbio.3002124.s017.docx]

RESPONSE TO REVIEWS:

Reviewer #1: This is an elegant and detailed description of cellular changes associated with Necrotizing enterocolitis (NEC) a devastating neonatal gastrointestinal (GI) complication that is associated with significant morbidity and mortality.

The authors have examined this hard-to-study population, using state of the art methods that include the scRNA seq, next gen TCR seq with some protein-level validation using assays like TUNEL and smFISH where appropriate.

Specifically, scRNAseq was performed on 11 subjects (6 NEC and 5 neonatal samples). To enable a precise estimation of population proportions, computational deconvolution of bulk RNAseq (10 NEC and 4 neonatal samples) data based on the clusters identified in the scRNAseq dataset was performed. Further, next generation sequencing (NGS) of TCRβ was performed to identify clonality changes associated with NEC (5 NEC and 9 neonatal samples). Finally, scRNAseq was combined with imaging mass cytometry (7 NEC and 3 neonatal samples) data to define niche and cellular interactions enriched in NEC.

Findings include numeric increase in various cell types in NEC including Macrophages.
Further, qualitative Mac associated changes included upregulation of key inflammatory programs. Similar features also seen in DCs. T cell subset changes showed an increased frequency of TRBV10 and reduced TCRb length with the possibility of an increase in CMV-6reactive T cell clones although the expression of IL17 was not detected in NEC. No significant change in Treg associated genes (rather a decrease in some of them) was noted. Other changes included numeric increase in lymphatic and vascular endothelial clusters with an upregulation of pro inflammatory programs and an increase in apoptosis and coagulation-related genes.

Epithelial cells changes were characterized by a reduction of villous-top APOA4+ cells and a relative increase in crypt-associated LGR5+ cells. Further, there was an increase in chemokines and Duox2+ expression by the epithelium. Increased interactions of endothelial cells with DCs and between hematopoietic and non-hematopoietic cell types was noted.

Overall, this is an impressive body of work using a number of high dimensional approaches. The main critique for such analyses is of course that this is descriptive. That said, it will serve as a useful and important resource for further studies into the pathogenesis and treatment of NEC.
As mentioned by the authors, distinctions between medical therapy responders- and non-responders would be important next steps as would be further analyses of the pathophysiological pathways, especially characterizing the key pathogenic events that lead to adverse clinical outcomes

**We very much appreciate the reviewer’s thoughtful comments and completely agree with the next steps in the field of NEC pathogenesis.**

Another critique of the paper is regarding the writing style- while the flow is good and the manuscript is easy to follow, the discussion ends up being repetitive and inordinately long in places. As one example, the discussion about a4b7-directed Vedolizumab is ill-informed due to the relatively long latency of action of the drug that would preclude its use in these critically ill neonates. There are other areas where the discussion could be focused and streamlined into a few critical areas of pathogenic mechanisms and their significance.

**We have re-written the discussion to be succinct and appropriately reflect important pathogenic mechanisms.**

Finally, given that the authors are positioning this as a potential resource paper, it would be important to also provide detailed sc cluster tables (not just the deconvolution analyses).

**We completely agree and have now added additional tabs to Supplementary Table 2 that include all of the markers of the relevant sub-clusters (myeloid, T/NK cells, endothelial, epithelial and fibroblasts).**

Altogether, my enthusiasm for this paper is very high barring the caveats mentioned above.


Reviewer #2: Major
1.      From Fig 1E, it would appear that the greatest change in cell type proportions in NEC is increased fibroblasts and decreased enterocytes. If we take these changes as pseudo-indicators of tissue injury, it indicates a variable level of tissue injury across the NEC samples in this dataset. If we assume that a 'normal' intestinal biopsy to be made up of mostly enterocytes as seen in the neonatal sample, NOT removing enterocytes and fibroblasts from 'all cells' would inevitably lead to observed changes in the proportions of other cell types. In other words, if both neonatal and NEC samples had 100 total cells, ~90 of these cells in the neonatal group would ALWAYS be enterocytes and ~10 would be non-enterocytes. Whereas in the NEC group, the non-enterocyte cell number could be highly variable purely due to a change to enterocyte numbers. In my opinion, it would be more appropriate to show the proportional change of enterocytes and fibroblasts as a fraction of all cells first and then for the rest of the cell types, look at the fractional change in the non-enterocyte/fibroblast fraction. This allows for a direct comparison between non-enterocyte proportions in neonatal and NEC samples.

**We have followed the reviewer’s suggestion and now present the relative proportions of non-fibroblasts and enterocytes while renormalizing over the set of cells that excludes enterocytes and fibroblasts. This is described on page 5:**

“Our atlas enabled exploring the detailed gene expression changes in distinct cell subsets. Furthermore, bulkseq analysis followed by computational deconvolution facilitated determination of major cluster abundances (**Fig 1E)** which revealed a substantial increase in the proportions of fibroblasts and a decrease in the proportions of enterocytes. Among the remaining cells, we idetentified a significant increase in the proportions of T/NK cells, vascular/lymphatic endothelial cells and a decrease in the proportions of DCs and B cells (**Fig 1E**, using False discovery rate (FDR)<0.25 as measure of significance).”

**And in the caption of Figure 1 (page 17):**

**“**Each dot is a sample, fractions of enterocytes and fibroblasts normalized to the sum of cell fractions, remaining fractions normalized to the sum of all cells after excluding fibroblasts and enterocytes;”

**New Figure 1:**

2.      Line 104: "This analysis revealed an increase in the numbers of..." Based on Fig 1E alone, it is not appropriate to say 'numbers' but rather proportion. Similar comment for Line 106: "...decrease in numbers..."

**We have now changed the phrasing as suggested. Now lines 103-108.**

3.      Line 118-119: It is not readily apparent how these genes are considered anti-inflammatory. The reference provided only potentially links TGFBI and anti-inflammatory macrophages.

**Our apologies for omission. We have added references for each of the genes listed in their roles in M2 like or anti-inflammatory macrophages. Now lines 120-122.**

4.      Figure 2G: It would appear that only 3 NEC infants had increased macrophage proportions in Fig 1E, with all other infants having what appears to be nil macrophages. Yet there are now >3 NEC infant with increased inflammatory macs in Fig 2G. How is this possible? Is it just that in 1E there are overlapping datapoints in Fig 1E?

**Figure 1E shows the proportions on a linear scale. Indeed, three of the samples have profoundly higher estimated levels of macrophages. Notably, however, the estimated proportions in the other samples are not 0, as can be seen when plotting the same data on a logarithmic scale:**

**Figure R1 – estimated cell proportions (previous Figure 1E) in linear scale (A) and logarithmic scale (B). Please note that we have changed the normalization in Figure 1E in the revised manuscript as suggested by the reviewer in point #1.**

**Figure 2G shows the breakdown of macrophage proportions among the different classes (inflammatory and anti-inflammatory macrophages). The relative proportions of these two subtypes varies, including in the NEC samples that have lower overall macrophage proportions.**

5.      Figure S2A: It is not clear how the genes shown in Fig S2A define the ILC subset. It is almost identical to activated T cells? Please elaborate here and for all other subclustering analyses on how cell types were labelled e.g. was it using signature genes from another study? Please reference.

**We have now elaborated on the systematic manner by which we have annotated our atlas clusters on page 30:**

“Clusters were annotated based on a previous comprehensive cell atlas of the human small intestine by Elmentaite et al. (88)(see **Supplementary Table 2** for a complete list of markers obtained using FindAllMarkers in Seurat). Specifically, the summed expression of the top 10 markers for each of our atlas clusters were examined over the clusters of the atlas of Elmentaite et al.(88) and the published cluster name with highest expression was assigned.”

An example of the annotation procedure can be seen in Figure R2:

**Figure R2 – Annotation procedure: A) Gut cell atlas of the human small intestine (Elmentaite et al.**^1^**) annotated by the assigned clusters in Elmentaite et al. (top left) and colored by the summed percent expression of the top 10 markers of the clusters in our study. B+C) Sub-clustering of Myeloid cells (B) and Epithelial cells (C) in Elementaite et al.**^1^ **colored by the summed percent expression of the top 10 markers of the clusters in our study.**

**The ILCs and activated T cells clusters are indeed very similar. In order to annotate the two clusters in a systematic manner, we pooled the ILC clusters from Elmentaite et al.**^1^ **and the activated T cells clusters and compared our marker expression between these two sets of cells. Figure R3A demonstrates that the top 10 markers of our ILCs cluster are indeed higher in the combined ILCs clusters compared to the combined activated T cells cluster in Elmentaite et al.**^1^ **Conversely, Figure R3B demonstrates that the top 10 markers of our activated T cell cluster are indeed higher in the combined activated T cell cluster compared to the combined ILCs cluster in Elmentaite et al.**^1^

**Figure R3- Ridge plot of the summed percent expression of top 10 ILCs markers (A) or activated T cells markers (B) taken from our study in the combined ILC and activated T cells clusters from Elmentaite et al.**^1^ **P-values were calculated using Wilcoxon ranksum test.**

**We have now also updated Figure S2A to include a subset of the markers systematically found by our annotation procedure. This highlights the distinct identities of the clusters:**

**New Figure S2:**

**Please note that the systematic annotation procedure we have now followed in the revision resulted in a revised annotation of the cluster previously annotated as ‘Tissue resident memory T cells’. We now find this cluster in fact overlaps the ‘Th1’ cluster in Elementaite et al.**^1^ **(See details in the response to reviewer comment 13).**

6.      Figure S2A: Color legend for average expression doesn't make sense given that the red color gradient starts from a negative value. How can average expression of the gene be a negative value?

**We used sctransform in seurat- sctransform calculates a model of technical noise in scRNA-seq data using ‘****regularized negative binomial regression’. The residuals for this model are normalized values, and can be positive or negative. Positive residuals for a given gene in a given cell indicate that we observed more UMIs than expected given the gene’s average expression in the population and cellular sequencing depth, while negative residuals indicate the converse.**

**We have now explained the scale presented (mean-transformed Seurat SCT transform) on page 20 (fig S2A captions):**

“Values are residuals of regularized negative binomial regression, log1p transformed, regressed to the sum of UMIs and centered.”

**And page 29+30:**

**“**Cells were normalized and scaled using the SCTransform function (residuals of regularized negative binomial regression, log1p transformed and centered), with regression of sum of UMIs (vars.to.regress = “nCount_RNA”).”

7.      Fig S2E: According to deconvolution, practically all of the bulk neonatal samples were naive T cells and yet in the sc-RNAseq in Fig 3B, naive T cells are the rarest T/NK/ILC cluster-type cell to be sampled? This does not make any sense as 3/4 neonatal control samples used in bulk RNA-seq was also used for sc-RNA-seq, suggesting they should be practically the same? Can the authors please comment on this discrepancy.

**This is an important point we now stress more in the revised manuscript. Single cell analysis does not reflect the true proportions in the tissue. This is due to the differential viability of different cells types to the isolation and sequencing protocol. The single cell data provides the expression signatures of distinct cell types, whereas the bulk RNAseq enables extracting true proportions. We now elaborate on this on page 5 and provide a relevant reference:**

**“**While single cell atlases are powerful for extraction of expression signatures, estimation of population proportions from such datasets can be skewed due to differential viability of extracted cells (10). To enable precise estimation of population proportions, we performed computational deconvolution of bulk RNAseq (6 NEC and 4 neonatal samples that passed threshold, Methods) data based on the clusters identified in the scRNAseq dataset (**Fig 1A**).”

8.      Figure 4L: It is not appropriate to perform statistical testing on repeated measurements sampled from 2 infants per group. Would suggest to remove 4L and state that J and K are representative images from n = 2 per group.

**We agree and have now removed 4L and added the statement for 4J,K in figure captions (page 18):**

“J and K are representative images from n=2 subjects per group.”

9.      Figure 5H: There are very few crypt cells in neonatal samples. By combining crypt + mid-bottom villus into a single group, there is a potential bias that any differentially expressed genes may really just be identifying the fact that there are more crypt cells in NEC than neonatal group. For example, are LCN2, REG1A, REG1B and DMBT1 crypt-associated genes? Please verify that DEGs here are not due to crypt-cell bias in NEC group or only do DEG analysis on crypt OR mid-bottom villus separately.

**We have now replaced Figure 5H to include only the mid-bottom villus zone: New Fig 5H below**

10.     Line 227: Please elaborate on what is included in the signature of TLR4 activation or the source of this signature.

**We have now included the list of genes in the Methods section on page 30-31:**

“TLR4 signaling pathway genes were taken from REACTOME pathway database: "TLR6","NOD1","TAB1","IRAK3","TIRAP","CHUK","CREB1","ATF2","MAPK14","TICAM1","AGER","DUSP3","DUSP4","DUSP6","DUSP7","ELK1","TAB2","FOS","LY96","PELI3","TAB3","TBK1","HMGB1","APP","TICAM2","IKBKB","IRAK1","IRAK2","IRF3","IRF7","JUN","PEDS-UBE2V1","MEF2A","MEF2C","MAP3K1","MYD88","ATF1","NFKB2","NFKBIA","NFKBIB","IRAK4","ECSIT","PPP2CA","PPP2CB","PPP2R1A","PPP2R1B","PPP2R5D","MAPK1","MAPK3","MAPK7","MAPK8","MAPK11","MAPK9","MAPK10","MAP2K1","MAP2K2","MAP2K3","MAP2K6","MAP2K7","PELI2","PELI1","SIGIRR","RELA","RPS6KA1","RPS6KA2","RPS6KA3","RPS27A","S100A12","S100B","SAA1","NOD2","MAP2K4","MAP3K7","BTK","TLR1","TLR2","TLR3","TLR4","TRAF3","TRAF6","RPS27AP11","UBA52","RPS27AP11","UBE2N","MAPKAPK3","IKBKG","RIPK1","RIPK2","RPS6KA5","MAPKAPK2","CD14","IKBKE","CDK1".”

11.     Line 229-230: Given this statement and my suggestions on Figure 5H, it would be interesting to see if the increase to the TLR4 signaling pathway is still observed when comparing only the mid-bottom villus fractions between NEC and neonatal given the more balanced comparison.

**We thank the reviewer for this important observation, indeed the significant difference stems from the difference in proportions. We now state this in the manuscript on page 10-11:**

**“**Notably, signature of TLR4 activation was enriched in NEC compared to neonatal epithelium (**Fig 5I**), consistent with previous reports of overactivation of the pathway in samples with NEC(23,30,48). This increase in TLR4 pathway activation (Methods) is likely due to an increase in the proportions of bottom and crypt villus zones, and indeed TLR4 pathway activation was not differentially expressed when stratifying for crpyt/mid-bottom enterocytes.”

12.     Line 303-305: As far as I understood, the interaction analysis did not provide direct evidence of interaction between these cell types but rather an indication that they may be interacting.

**We have now rephrased the sentence (page 13):**

**“**NLR and TLR activation have been implicated in NEC pathogenesis both in human disease and in murine models, with the current study providing a context that macrophages are responding to these signals(23–25) and may be interacting with both immune and non-immune cells within the small intestine.”

13.     Line 308-313: Given the interest to the field of the role of Th polarization and its links to NEC pathogenesis, have the authors tried to determine if the activated T cells have a predominant Th signature of any kind? Or do they only detect some upregulation of Th17 genes but NOT that of Th1 and Th2?

**We thank the reviewer for this comment. We have now systematically reannotated our atlas cell clusters based on the published human gut cell atlas of Elmentaite et al.**^1^**, as described on page 30 and in the response to reviewer comment 5. Upon this procedure, the annotation of all clusters remained unchanged, with the single exception of the cluster previously annotated as Tissue resident memory T cells (TRMs). We found that the highest markers of this cluster in fact overlap with Th1 (Figure R4A, red arrow). Figure R4B shows the summed expression of the top 10 Th1 markers taken from Elmentaite et al.**^1^ **over the clusters in our atlas, further supporting the re-annotation of the Th1 cluster. We further examined known markers of Th2 (taken from Danaher et al.**^2^**, these cells do not appear in Elmentaite et al.). We found that highest expression of these Th2 markers was in the proliferating T cells cluster and did not seem to be high in any other cell type (Figure R4C).**

**Figure R4 – A) Summed percent expression of the top 10 markers in our data of the cluster previously annotated as TRMs shown on Elmentaite et al.**^1^ **data. Th1 cluster marked in red arrow. B) Summed percent of the top 10 Th1 cells markers extracted from Elmentaite et al.**^1^ **shown over the clusters in our study. C) Summed percent of the Th2 cells markers extracted from Danaher et al.**^2^ **shown over the clusters in our study.**

14.     Given the connection between using the word 'significant' has to being statistically significant, the authors need to more accurately describe their results when it is NOT statistically significant as trends. For example, Line 157-168, none of these are statistically significant.

**We agree and have now rephrased (page 8), please note we present q-values (FDR corrected p-values) and therefore still describe the changes with q<0.25 as significant as follows:**

“Computational deconvolution analysis of the bulk RNAseq data indicated a non-significant increase in activated T cells, and a significant increase in Th1 cells and proliferating T cells and a decrease in Naïve T cells (**Fig S2E**, using FDR<0.25 as measure of significance).”

Minor
1.      Figure 1: Visualization of 1E should be improved to be able to see datapoints clearly (for example, using more horizontal space where dots overlap or transparency or dot size). For example, in Fibroblasts NEC group there should be 10 datapoints but it looks like there is at most 6 dots there.

**We have now changed accordingly to improve clarity (see updated figure 1 on page 3). In the panel we have only included samples for which the Spearman correlation between the observed and deconvolution-based reconstructed expression where above 0.3. We note this in the caption of figure 1E, 2G,5F and S2E:**

“Only samples with Spearman correlations > 0.3 between the mixture data and the synthetic mixtures are shown (neonatal: n=4, NEC: n=6).”

**In addition, it is explained in the methods and clarified in in the text on page 5:**

**“**To enable precise estimation of population proportions, we performed computational deconvolution of bulk RNAseq (6 NEC and 4 neonatal samples that passed threshold, Methods) data based on the clusters identified in the scRNAseq dataset (**Fig 1A**).”

2.      Figure S4B, color legend missing.

**We have now corrected this:**

3.      Supplementary Table 3: Is the 'Ratio' column referring to Log2 fold-change? Suggest to rename to be clear. If not, what does it mean to have a Ratio of 0.95? Is it 5% decreased in NEC vs neonatal?

**We have clarified that this is the actual ratio and replaced with “NEC/Neonatal” in the table column header. Indeed, 0.95 means a 5% decrease in NEC compared to Neonatal.**

4.      Supplementary Table 5: There are several antibodies that are labelled with 'Harvard Core' for source. Please provide original source unless these antibodies are available only from Harvard Core.

**We have updated this table to appropriately reflect the correct antibody vendor.**

5.      Would recommend that n numbers are described in figure legends.

**We have now added this data to the relevant figure legends.**

6.      Line 200: There is no direct evidence shown in FigS3D-E that there is a correlative relationship between TNF/IL1B with SELE and ICAM1, e.g. the more TNF there is, the more SELE is present. The relationship is associative.

**We have rephrased as suggested by the reviewer (page 9):**

“Both *IL1B* and *TNFA* were enriched in the NEC inflammatory macrophages and were associated with an increase in *SELE* and *ICAM1* in the activated endothelium in NEC (**Fig S3C-D)**.”

7.      Line 203: Please elaborate on what LYVE-1+ cells are. What is the purpose of LYVE-1+ staining? Endothelial cell marker?

**LYVE-1 staining here has been used to identify endothelial cells.**

8.      Line 240: First mention of the word IMC, unclear what that is.

**We have now defined Imaging Mass Cytometry as IMC.**

9.      Line 253: Unclear what a mf cell is.

**We have now corrected to “macrophages” or “Mφ”.**

10.     Line 567-568: What does it mean that only edge piece was obtained for intestinal tissue acquisition? Is this a biopsy that contains both macroscopically NEC-afflicted inflamed tissue and non-inflamed tissue? More detail if possible should be provided for example, on the exact site of sample collection in NEC, i.e. Ileal, jejunal?

**The tissue biopsy was obtained from the leading edge away from the site of intestinal necrosis. Samples had macroscopically inflamed tissue. The wording was update in the manuscript.**

11.     Line 588, what is T-Cell Media? Please provide more details.

**We have clarified the components of T-cell media in the page 24:**

**“**Single cell suspension obtained from small intestine tissue digestion was washed in T-Cell Media (RPMI medium plus 10% FBS (Corning), 1X GlutaMax, 10mM HEPES, 1X MEM NEAA, 1 mM sodium pyruvate, 100 I.U/mL penicillin and 100 μg/mL streptomycin) twice followed by enrichment for live cells with the dead-cell removal kit using the MACS Cell separation system (Miltenyi kit # 130-090-101).”

12.     Line 601: What was quality controlled? I assume RNA quality, and what was the result? For example, was RIN over 8 for all samples?

**Yes, RNA was quality controlled per company guidelines and all smaples sequenced passed the company guidelines. We have updated this in the methods on page 25:**

**“**RNA quality control (QC) was completed by MedGenome via Qubit Fluorometric Quantitation and TapeStation BioAnalyzer per company guidelines, with all samples passing the QC.”

13.     Line 607: Fixed for how long? How was it processed before being embedded in paraffin or was it directly taken out of formalin into paraffin?

**In updated methods section page 25:**

“Biopsy-sized pieces of small intestinal tissue were fixed in formalin for 48 hours on day of collection, transferred to ethanol and subsequently embedded in paraffin in batches.”

14.     Line 610: Is this R&D systems?

**Yes, this has been updated.**

15.     Line 614: No company reference provided for LipoR-Ln115 and Ir-intercalator

**We have updated the text to reflect the appropriate supplier.**

16.     Line 724: "Clusters were annotated based on markers from literature" but no references are indicated.

**Please see our detailed response in comments 5,13 (Major). We have now reannotated all of our clusters systematically, as described above.**

17.     Line 728-730: Is this a repeat of Line 720-723?

**Indeed, the sub-clusters underwent the same pre-processing, however we feel it is important to leave this text as is in the Methods to maintain the maximal transparency and facilitate reproducibility of the data analysis.**

18.     There are a few instances of inconsistent unit labelling and likely exporting errors to watch out for. E.g. Line 581: ML, Line 609: uM, Line 199, Line 200

**We have updated these in the text to reflect the correct labeling.**

Reviewer #3: In this manuscript, combining scRNA-seq and other analyses, Egozi et al depicted a comprehensive atlas of the human gastrointestinal complication, necrotizing enterocolitis (NEC), laying a good foundation for further understanding of the disease. However, some issues need clarification for the publication in PLOS Biology.

1.      Through the deconvolution method, the authors showed that the two kinds of myeloid cells, macrophages and dendritic cells are increased and decreased, respectively, in NEC (Fig. 1E). However, inflammatory genes were increased in macrophages and dendritic cells, and some reports that the authors referred to also revealed an enrichment of myeloid cells in NEC. Then, how to explain the decrease of dendritic cells and the increase of their inflammatory genes?

**We have now clarified these findings in the text. Our data indeed indicates that there is an overall reduction in the proportions of DCs (in the revision we re-normalize to non-enterocyte and fibroblasts and find that the reduction in DC proportion indeed remains). However, in terms of the gene expression signatures of the DCs, they exhibit changes, including increased expression of pro-inflammatory genes. We have clarified this in the text on page 6-7:**

**“**Notably, although the fraction of DCs in NEC was lower than in neonatal samples, their expression signatures showed elevation of pro-inflammatory genes, similarly to the NEC macrophages.”

2.      Some descriptions seem abrupt and lacked logicality. For example, the authors performed a search of the public database to determine the expanded clones in NEC, but they directly described the sequence known to bind to CMV without any explanations.

**The two CMV clones were the only known clones that were significantly enriched in NEC. We have now clarified this point on page 7-8:**

**“**The majority of the top clones observed in NEC were previously reported public clones. Our search revealed only three public clones that were significantly enriched in NEC. Interestingly, the amino acid sequences of two of these clones had an identical sequence to clones known to bind to cytomegalovirus (CMV) (CMV-1, CMV-2) (**Fig S2D, Supplementary Table 4**).”

3.      In Fig. 4, the authors showed that there were more apoptotic endothelial cells in NEC, which is inconsistent with the increased number of endothelial cells as revealed in Fig. 1E. The apoptosis level is also in contrast to the high expression of pro-inflammatory signatures.

**This is an interesting point, we now show that the elevated proportions of endothelial cells could be a result of increased proliferation of these cells in NEC, balancing their elevated death rates. This is described on page 9-10:**

“Our computational deconvolution suggested an overall increase in the proportions of vaslcular/lymphatic endothelial cells (**Fig 1E**). In line with the elevation in apoptotic markers, the elevated cell proportions could be a result of compensatory elevated proliferation, balancing the elevated cell death. Indeed, as 4.4% vs. 0.7% of vascular/lmphatic cells in NEC/neonatal respectively were positive for the mRNA of the proliferation marker Mki67 (Fisher exact test p=0.0032). “

4.      In Fig. 5D and 5E, the authors performed staining of villus top and bottom genes using normal tissues, and the NEC tissues should also be included to validate the loss of villus-tip enterocytes.

**We have included imaging mass cytometry (IMC) validation that demonstrates the profound villus blunting in Fig. 5G.**

5.      The authors declaimed that this work help to understand NEC pathogenesis. However, the relative information is lacking.

**We have now rephrased this in the Discussion on page 13:**

**“**The pathogenesis of NEC remains poorly understood and to date, few studies utilize single cell approaches to describe the altered interactions among various intestinal cells(9). Here, we systematically explored the changes in cell proportions and expression signatures in NEC using single cell approaches.”

6.      While inflammatory changes were found in NEC samples, are there any differences within NEC groups? Are there any relationships between gestational/postnatal age and inflammation severity?

**We have performed DGE between the bulk NEC samples stratified by gestational age at birth (group A- subjects born at <= 28 gestation age (GA) weeks, group B- subjects born at > 28 GA weeks). We found very few differences (only 15 genes significantly different between the two age groups, Figure R5A). Importantly, the elevation of inflammatory genes seems to be independent of the NEC age at birth (Figure R5B):**

**Figure R5 – A) Volcano plot demonstrating very little difference in gene expression between the two GA groups - NEC group 1 (born at <= 28 GA weeks, n=5) and NEC group 2 (born at > 28 GA weeks, n=4). Red dots are significant genes with ratio>2 and false discovery rate corrected q-value<0.2. B) Representative inflammatory genes, which exhibit similar expression between the NEC groups and higher expression compared to the Neonatal samples (n=4). Values are log10(sum-normalized expression).**

7.      ScRNA-seq data are sufficient to yield enough information about cluster change. What is the reason for choosing bulk seq-based computational deconvolution for this purpose? Do their two methods give different results?

**This is an important point we now stress more in the revised manuscript. Single cell analysis does not reflect the true proportions in the tissue. This is due to the differential viability of different cells types to the isolation and sequencing protocol. The single cell data provides the expression signatures of distinct cell types, whereas the bulk RNAseq enables extracting true proportions. We now elaborate on this on page 5 and provide a relevant reference:**

**“**While single cell atlases are powerful for extraction of expression signatures, estimation of population proportions from such datasets can be skewed due to differential viability of extracted cells (10). To enable precise estimation of population proportions, we performed computational deconvolution of bulk RNAseq (6 NEC and 4 neonatal samples that passed threshold, Methods) data based on the clusters identified in the scRNAseq dataset (**Fig 1A**).”

8.      There are some grammar and spelling mistakes, e.g, "This is correlates with" (line 60), "Intrestingly" (line 153), etc.

**We have updated the text throughout.**

**References:**

1.	Elmentaite, R. *et al.* Cells of the human intestinal tract mapped across space and time. *Nature* **597**, 250–255 (2021).

2.	Danaher, P. *et al.* Gene expression markers of Tumor Infiltrating Leukocytes. *J. Immunother. Cancer* **5**, 18 (2017).
